# Copper Resistance Mediates Long-Term Survival of *Cupriavidus metallidurans* in Wet Contact With Metallic Copper

**DOI:** 10.3389/fmicb.2020.01208

**Published:** 2020-06-03

**Authors:** Laurens Maertens, Ilse Coninx, Jürgen Claesen, Natalie Leys, Jean-Yves Matroule, Rob Van Houdt

**Affiliations:** ^1^Microbiology Unit, Interdisciplinary Biosciences, Belgian Nuclear Research Centre (SCK CEN), Mol, Belgium; ^2^Research Unit in Microorganisms Biology (URBM), Narilis Institute, University of Namur, Namur, Belgium

**Keywords:** copper, *Cupriavidus*, drinking water, VBNC, heavy metal resistance

## Abstract

Metallic copper to combat bacterial proliferation in drinking water systems is being investigated as an attractive alternative to existing strategies. A potential obstacle to this approach is the induction of metal resistance mechanisms in contaminating bacteria, that could severely impact inactivation efficacy. Thus far, the role of these resistance mechanisms has not been studied in conditions relevant to drinking water systems. Therefore, we evaluated the inactivation kinetics of *Cupriavidus metallidurans* CH34 in contact with metallic copper in drinking water. Viability and membrane permeability were examined for 9 days through viable counts and flow cytometry. After an initial drop in viable count, a significant recovery was observed starting after 48 h. This behavior could be explained by either a recovery from an injured/viable-but-non-culturable state or regrowth of surviving cells metabolizing lysed cells. Either hypothesis would necessitate an induction of copper resistance mechanisms, since no recovery was seen in a CH34 mutant strain lacking metal resistance mechanisms, while being more pronounced when copper resistance mechanisms were pre-induced. Interestingly, no biofilms were formed on the copper surface, while extensive biofilm formation was observed on the stainless steel control plates. When CH34 cells in water were supplied with CuSO_4_, a similar initial decrease in viable counts was observed, but cells recovered fully after 7 days. In conclusion, we have shown that long-term bacterial survival in the presence of a copper surface is possible upon the induction of metal resistance mechanisms. This observation may have important consequences in the context of the increasing use of copper as an antimicrobial surface, especially in light of potential co-selection for metal and antimicrobial resistance.

## Introduction

The historical use of copper (Cu) for sterilization of drinking water and the treatment of various illnesses dates back to the third millennium B.C. Indeed, ancient civilizations ranging from Egyptian over Greek to Aztec utilized Cu in different formulations to combat bacterial proliferation in diverse conditions (Dollwet and Sorenson, 1985). Recently, the widespread use of antibiotics and concomitant development of antibiotic resistance mechanisms has brought renewed interest in the antimicrobial properties of Cu (Grass et al., 2011).

Copper metal, which is routinely used for plumbing works, can impart bactericidal effects on waterborne microorganisms via the leaching of Cu ions. The solubility of Cu in water depends on chemical parameters such as pH, concentration of dissolved oxygen and free Cl^–^ ions (Pehkonen et al., 2002), and the presence of chelating agents (Karczewska and Milko, 2010). For many bacterial species, relatively low concentrations of ionic Cu are sufficient for inactivation, e.g., *Legionella pneumophila* in drinking water shows a 6-log_10_ decrease in CFU/ml within 3 h after the addition of 100 μg/l (0.74 μM) CuCl_2_ (Lin et al., 1996), more than 99.9% of *Pseudomonas aeruginosa* cells in deionized water are inactivated within 24 h by adding 1 μM (160 μg/l) CuSO_4_ (Dwidjosiswojo et al., 2011), and hospital isolates as well as control strains showed near-complete killing after 48 h storage in a Cu vessel containing a saline solution or water (Cervantes et al., 2013). In addition to the toxic effects of ions present in the medium, the direct interaction of bacterial cells with the metal surface can lead to a strongly enhanced killing effect (Mathews et al., 2013), coined as contact killing and recently reviewed by Vincent et al. (2018). This enhanced toxicity was demonstrated by covering the copper surface with an inert polymeric grid, which prevented direct bacterial attachment but not the release of Cu ions in the medium, resulting in a drastic decrease in toxicity. Contact killing rates depend on, among else, copper content, temperature and humidity (Faúndez et al., 2004; Michels et al., 2009), and medium composition (Molteni et al., 2010). The mechanisms of Cu toxicity to bacterial cells have been under increasing scrutiny (Lemire et al., 2013; Vincent et al., 2018). Several modes of action have been put forward, although their sequence and interactions remain unclear. The production of reactive oxygen species can be catalyzed by Cu via a Fenton-like reaction *in vitro*, but contrasting data have been reported regarding its role in DNA degradation *in vivo* (Macomber et al., 2007; Tian et al., 2012; Warnes et al., 2012; Cui et al., 2014; San et al., 2015). The inactivation of Fe–S proteins by competition of Cu with Fe in an oxygen-independent manner has been reported (Macomber and Imlay, 2009; Arguello et al., 2013), as well as membrane damage via lipid peroxidation (Espirito Santo et al., 2011; Hong et al., 2012; Santo et al., 2012). In addition, the induction of a viable but non-culturable (VBNC) cell state after exposure to Cu has been observed in multiple studies. This state is defined as cells with low metabolic activity that maintain membrane integrity and a low level of gene expression, but which do not form CFUs on culture media (Davey, 2011; Schottroff et al., 2018). The VBNC state is thought to enable an increased resistance to many environmental stresses, including antibiotics (Orman and Brynildsen, 2013), oxidative stress and metals such as copper (Nowakowska and Oliver, 2013). Many questions still surround the VBNC state, but it is clear that VBNC cells can still pose problems since resuscitation and subsequent toxicity of bacteria from drinking water have been reported (Lee et al., 2007; Dwidjosiswojo et al., 2011; Li et al., 2014; Dopp et al., 2017; Ye et al., 2020).

While the inactivation of bacteria by low Cu concentrations might imply the absence of a Cu requirement for bacterial metabolism and growth, this is clearly not the case. In fact, bacteria must carefully regulate intracellular concentrations of this micronutrient and have evolved a multitude of mechanisms devoted to this task. In contrast to export systems, import of Cu to the cytoplasm is poorly understood, with early research indicating the importance of outer membrane porins in *Escherichia coli* (Lutkenhaus, 1977). In more recent studies, putative roles for major facilitator superfamily (Balasubramanian et al., 2011) and TonB-dependent transport systems (Ekici et al., 2012) have been identified. Free Cu in the cytoplasm is quickly bound by chaperones and delivered to export systems, such as P_IB__1_-type ATPases and HME-RND systems (Arguello et al., 2013). Multicopper oxidases with various functions provide an extra layer of defense. Some bacteria harbor multiple copper resistance mechanisms that can interact to a high level of complexity and lead to the ability to withstand millimolar concentrations. The *Cupriavidus metallidurans* species, isolated on many occasions from harsh metal-contaminated, oligotrophic industrial environments, provides a striking example. Type strain *C. metallidurans* CH34, which has become a model strain for studying metal resistance, was isolated in 1978 from a decantation basin in a non-ferrous metallurgical plant (Mergeay et al., 1978). Its *cop* cluster, located on the megaplasmid pMOL30 and conferring high resistance to Cu ions, consists of 21 genes among which several chaperones, a P_IB__1_-type ATPase, a HME-RND system and a multicopper oxidase. Almost all are upregulated after exposure to Cu^2+^ (Monchy et al., 2006, 2007). Remarkably, this species has been repeatedly found to contaminate the International Space Station, including on dust and in cooling and drinking water reservoirs (Pierson, 2001; Ott et al., 2004; Mora et al., 2016).

The importance of copper resistance mechanisms in relation to contact killing has only been poorly studied, especially in conditions relevant to drinking water treatment and in timeframes longer than several hours. In dry and semi-dry conditions, the deletion of copper resistance determinants leads to a slightly faster inactivation of *E. coli* (Espirito Santo et al., 2008), *P. aeruginosa* (Elguindi et al., 2009), and *Enterococcus hirae* (Grass et al., 2011). In this study, we investigated the inactivation kinetics of *C. metallidurans* CH34 in contact with metallic copper in drinking water and evaluated the role of its copper resistance mechanisms.

## Materials and Methods

### Bacterial Strains, Media and Culture Conditions

*C. metallidurans* CH34 (Mergeay et al., 1978), AE104 (plasmidless derivative of CH34) (Mergeay et al., 1985) and *Cupriavidus campinensis* AE1239 (derivative of DS185 carrying a Cu-responsive *lux*-fusion in pMOL90:Tn*4431*) (Collard et al., 1994) were routinely grown in Tris–buffered mineral medium (MM284) (Mergeay et al., 1985) supplemented with 2 g/l gluconate, on an orbital shaker at 180 rpm in the dark at 30°C. MM284 agar plates were prepared by adding 2% agar (Thermo Scientific, Oxoid, Hampshire, United Kingdom) to the liquid medium. Ionic Cu was added to the medium as CuSO_4_ (Sigma-Aldrich, Overijse, Belgium). Phosphate-buffered saline solution (PBS) was prepared by dissolving a PBS tablet (Gibco^TM^) in Milli-Q water (Merck Millipore, Belgium), achieving final concentrations of 10 mM sodium phosphates (to attain a pH of 7.4), 2.68 mM KCl and 140 mM NaCl. The solution was sterilized by autoclaving at 121°C for 15 min.

### Preparation and Setup of Survival Experiments

Copper (99,99%) and stainless steel (AISI 304) plates were prepared by submerging them in a 70% ethanol solution for 10 min to inactivate possible contaminants and to solubilize organic components on the plate surface. After this step the plates were sonicated at 130 kHz in a TI-H-5 (Elma Schmidbauer GmbH, Germany) in deionized water for 10 min to remove contamination and to dissolve any extant salts. Plates were then sterilized at 121°C for 20 min to kill any remaining cells, dried at 60°C and stored in sterile containers at room temperature. Bacterial cultures of *C. metallidurans* CH34 and AE104 were grown in MM284 for 72 h. While the stationary phase is reached in about 36 h, cells were cultured for an additional 36 h to induce a starvation response. This step was introduced to reflect natural environments and to diminish the confounding effects of sudden nutrient depletion in the main experiment. This approach did not affect the initial number of CFUs. Pre-induced *C. metallidurans* CH34 cultures were prepared by adding 300 μM CuSO_4_. Cultures were washed twice in filter-sterilized mineral water (Ordal, Ranst, Belgium) and subsequently resuspended in this mineral water (final OD_600_ of 0.1). The prepared Cu and stainless steel plates were separately inserted into a 50 ml conical centrifuge tube with vent cap until stuck against the conical tube bottom. No plates were inserted in the control condition tube. Forty ml of prepared bacterial suspension was added to each tube and all tubes were placed upright on an orbital shaker at 150 rpm in the dark at 30°C. A generalized overview of this setup can be found in Supplementary Figure 1. In a separate experiment, cultures were prepared identically, but instead of inserting a Cu plate, 10 μM CuSO_4_ was added. An equivalent volume of Milli-Q water was added to the control condition. All survival experiments were performed in biological triplicate.

### Viable Counts and Flow Cytometry

Samples for viable counts and flow cytometry were taken at 0, 1, 3, 5, 24, 48, 72, 96, 144, 168, and 192 h after the start of the experiment. For each sample, 20 μl of bacterial suspension was taken from ca. 1 cm below the water level. Cell enumeration by total viable count was performed by spreading 100 μl of a serial 10-fold dilution in sterile PBS on MM284 agar and counting colonies after a minimum of 4 days at 30°C. Cell enumeration by flow cytometry was performed by diluting 20 μl of bacterial suspension 100- and 1000-fold in filter-sterilized (0.2 μm) mineral water (Evian). Next, SYBR Green (Life Technologies) and propidium iodide (PI, Merck) were added to a final concentration of 1X (starting from a 10,000X commercial stock solution) and 200 μM, respectively. Suspensions were incubated in the dark at 37°C for 20 min to allow complete binding of the dyes. Stained bacterial suspensions were analyzed on an Accuri C6 (BD, Erembodegem) with a blue (488 nm, 20 mW) laser, which was calibrated according to the manufacturer’s recommendation. Standard optical filters included FL-1 (530/30) and FL-3 (670 nm LP). SYBR Green was detected with FL-1, PI with FL-3. Samples were analyzed using the BD Accuri C6 software version 1.0.264.21, and gating and counting of events was performed using the PhenoFlow package for R (Props et al., 2016).

### Biofilm Visualization

After 9 days of incubation, the copper and stainless steel plates were placed in a 6-well plate and immediately stained with 100 μl SYBR Green (1X) and propidium iodide (200 μM) solution. The plates were incubated in the dark at 37°C for 20 min. Unattached bacterial cells and excess dye were gently rinsed off the plates with 1 ml of PBS.

An automated inverted fluorescence microscope (TE2000-E; Nikon, Tokyo, Japan) equipped with a motorized XYZ stage, emission and excitation filter wheels, shutters and a triple dichroic mirror (436/514/604) was used for the image acquisition of the surface of the stainless steel and Cu plates. Images were acquired with a 20X objective and an Andor iXon EMCCD camera (Andor Technology, South Windsor, CT, United States). For each sample, at least five sets of 25 pictures in a 5 × 5 grid, each containing one field of view, were computationally stitched together with 15% overlap using NIS-elements AR 5.11.01 (Nikon).

### ICP–MS

At 0, 5, 48, and 216 h after the start of the experiment samples were taken for ICP–MS measurement to determine the concentrations of Cu, Ni, and Zn in the aqueous phase. Two milliliters were centrifuged at 10,000 *g* for 2 min, and the supernatant was stored at −20°C until further processing. Samples were acidified to a final concentration of 2% HNO_3_ and metal concentrations were measured with an ICP–MS X-Series II (Thermo Fisher Scientific).

### Biosensor Experiments

The response of the Cu biosensor AE1239 was quantified using a multimode microplate reader (CLARIOstar^®^, BMG Labtech). This strain has a reported detection limit of 1 μM (Kanellis, 2018). AE1239 cultures were prepared by growing them in MM284 for 72 h at 30°C, subsequently washing them twice with filter-sterilized mineral water (Ordal) and resuspending them in either filter-sterilized mineral water or MM284. The response on metal plates was measured by pipetting 200 μL of cell suspension on top of each plate placed in a six-well plate. Focal height was optimized to 7.5 mm, and a maximal gain of 4095 was used. Measurements were performed approximately every 5 min without intermittent shaking of the plate. The response to ionic Cu was measured by adding 200 μL to a 96-well plate. Focal height was optimized to 11 mm, again with maximal gain. For these experiments, intermittent shaking occurred at 150 rpm and the optical density at 600 nm was measured in addition immediately before the luminescence measurement.

### Statistics

For statistical analysis, the lmerTest and emmeans-packages in R 3.6.1 were used to fit linear mixed models (lmer), followed by a pairwise comparison and Bonferroni’s procedure for multiple testing [pairs(emmeans)]. Adjusted *p*-values below 0.05 were considered statistically significant.

## Results and Discussion

### Survival of *C. metallidurans* CH34 in Wet Contact With Metallic Cu

**FIGURE 1:**
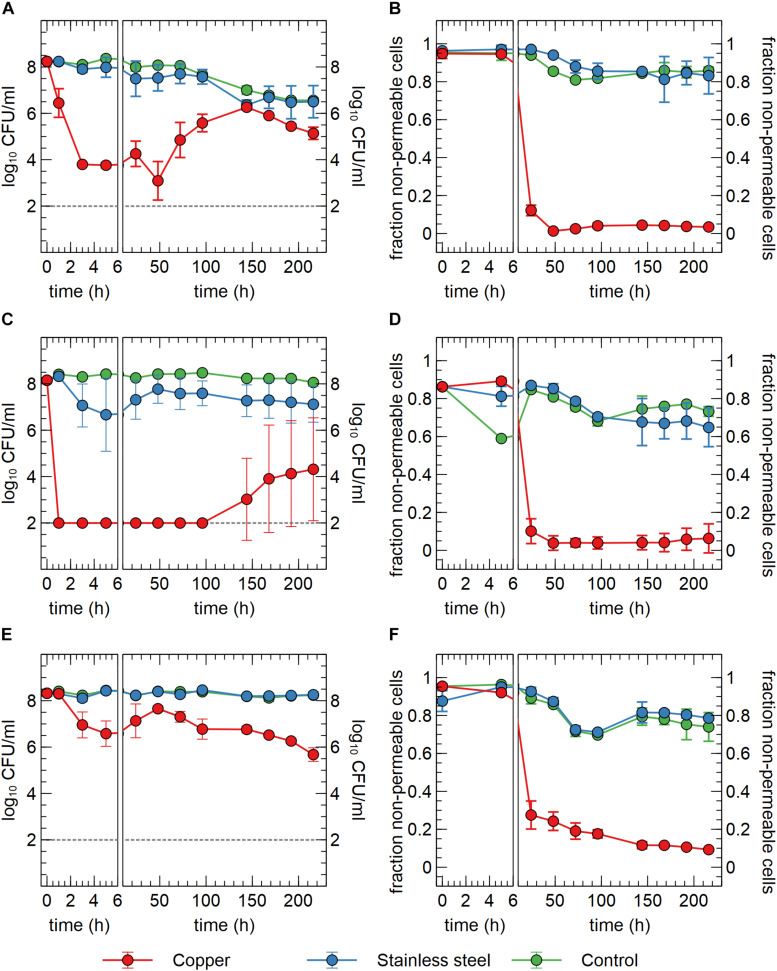
Viable counts **(A,C,E)** and fraction of non-permeable cells **(B,D,F)** of *C. metallidurans* CH34 **(A,B)**, *C. metallidurans* AE104, lacking metal resistance mechanisms **(C,D)** and *C. metallidurans* CH34 pre-induced with 300 μM CuSO_4_ **(E,F)** in the presence of metallic Cu (red), stainless steel (blue) and control condition (green). All experiments were independently performed with *n* = 3.

In a first setup, we tested the survival of *C. metallidurans* CH34 in sterilized drinking water in contact with either a submerged Cu plate, a stainless steel plate or no plate. The Cu condition showed a clear and significant drop in viable counts of more than 4-log_10_ within 3 h after the start of the experiment (Figure 1A). This decrease in viable count was confirmed but not exacerbated after 5 h. Between 5 and 48 h, the viable count remained stable, after which it increased significantly with 2.7-log_10_ until 144 h after the start of the experiment. After 144 h, viable counts decreased again, but this process was concomitant to the decrease observed in the stainless steel and the control condition, where some loss of viability was measured over the course of the experiment (Figure 1A). These results evoke the question whether the decrease in viable counts is due to cell death, sublethal injury or a rapid conversion to a VBNC state. Since our experimental setup cannot discriminate between sublethally injured and VBNC cells, all cells with a non-permeable membrane (flow cytometry analysis) that do not form CFUs are regarded as VBNC cells. In case of an initial killing of cells, the surviving 0.01% of cells could utilize the scarce assimilable organic carbon (AOC) in the drinking water and the contents of permeabilized cells to mount a defense against the toxic effects of Cu, after which regrowth could be possible. In the second case, VBNC cells could use AOC, as well as intracellular stored nutrient reserves such as polyhydroxybutyrate, to repair cellular damage and activate Cu resistance mechanisms, after which a gradual resuscitation to a culturable state is achieved without any change in cell number due to regrowth. It is conceivable that both scenarios occur in parallel.

**FIGURE 2:**
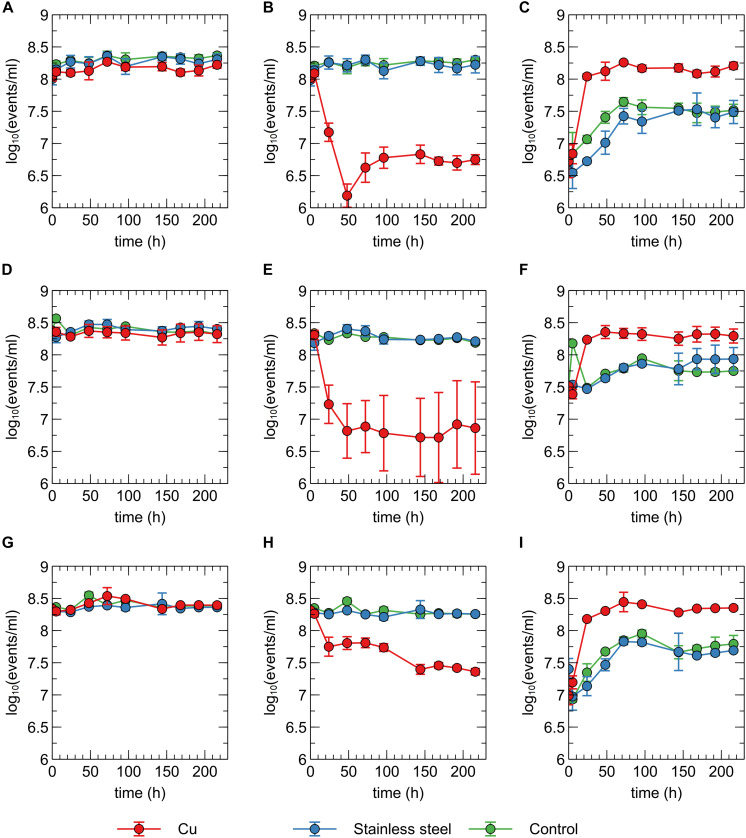
Concentration of total events **(A,D,G)**, events corresponding to non-permeable cells **(B,E,H)** and events corresponding to permeable events **(C,F,I)** recorded for *C. metallidurans* CH34 **(A,B,C)**, *C. metallidurans* AE104 **(D,E,F)** and *C. metallidurans* CH34 pre-induced with 300 μM CuSO_4_ **(G,H,I)** in the presence of metallic Cu (red), stainless steel (blue) and control condition (green). All experiments were independently performed with *n* = 3.

In addition to viable count data, cell viability was assessed via SYBR Green+PI staining and flow cytometry. SYBR Green was used to stain all cells as it can enter cells independent of their physiological state. The total number of all events per ml did not significantly vary between conditions and over time (Figure 2A), indicating that no considerable cell disintegration or sedimentation occurred, and that VBNC cells could be detected by the flow cytometry approach. Over the course of several days, recorded events in the Cu condition showed a decrease in their SYBR signal strength, while the PI signal became stronger (Figures 1B, 2B,C and Supplementary Figure 2). Thus, as visualized in FL3 vs FL1 plots (Supplementary Figure 2), events migrated from the original zone of non-permeable cells to a zone that contained permeable (likely non-viable) cells, which coincided with the positive control of heat-killed cells. The latter represented the gradual decay of the cell envelope, allowing more PI to enter the cells. As a result, the number of events corresponding to non-permeable cells decreased with 2-log_10_ after 48 h after which they remained fairly stable around 5 × 10^6^ events per ml (Figure 2) and this trend was inversely proportional to the number of events corresponding to permeable cells. Finally, VBNC cells accounted for the majority of the viable cells after 24 to 48 h (roughly 99.9 and 99%, respectively) and gradually decreased to roughly 75% at 144 h.

The discrepancies between the viable and flow cytometry counts merit in-depth discussion. The lag of several hours between the initial decrease in cell culturability and the decrease of events in the non-permeable zone could be due to purely physicochemical processes in which several hours are needed before the cell envelope of killed/injured cells is sufficiently permeable to PI. Alternatively, it could be due to a rapid conversion to a VBNC state in which cells are not permeable to PI since their envelope is still intact. As such, culturable cells cannot be distinguished from VBNC cells by our flow cytometry analysis. Second, no clear increase of events corresponding to non-permeable cells was observed, which indicates that cells are recovering from a metabolic state that can only be measured via culturing, instead of regrowth of the population after an initial killing. Therefore, we hypothesize that within 48 h, ca. 5% of the initial population is converted to a VBNC cell state, while the remaining 95% of the population is permeabilized and thus killed. Almost 25% of these VBNC cells recover to a culturable state after 144 h. Finally, although PI is an indicator for cell viability and should only stain cells with damaged or permeable membranes (Strauber and Muller, 2010), several studies have reported contrasting data regarding the use of PI for viability staining, e.g., when applied in the early exponential phase (Shi et al., 2007). For instance, ostensibly PI-permeable *Shewanella decolorationis* S12 cells, supposedly in a VBNC state, have been observed to repair membrane damage and lose PI signal strength upon switching of their respiratory metabolism (Yang et al., 2015). This complex relation between PI staining and the presence of intermediate cell states has been reported for several other bacterial species (Berney et al., 2007). Nonetheless, we opted for a SYBR Green+PI staining procedure as it is routinely used in viability testing. To summarize, we conclude that *C. metallidurans* CH34 is indeed being killed by the presence of the Cu plate, but to a much lesser extent than indicated by the viable count as the Cu plate induces a VBNC cell state in part of the population.

### Copper Resistance Mechanisms Provide a Defense Against Metallic Copper

We hypothesized that the induction of copper resistance mechanisms allowed for either growth or resuscitation of copper-stressed cells. To test this hypothesis, two additional experiments were performed. In the first one, a similar setup was used with strain *C. metallidurans* AE104. This latter is derived from CH34 by curing of its megaplasmids pMOL28 and pMOL30 that encode most functional copies of its metal resistance mechanisms, including the main copper resistance determinants on pMOL30, as detailed in the introduction. Consequently, its minimum inhibitory concentration (MIC) is much lower than that of CH34 for most metal ions. Immediately apparent was the significant 6-log_10_ decrease in viable counts after only 1 h in the Cu condition, which further decreased to the detection limit after 3 h (Figure 1C). It is evident that the lack of metal resistance mechanisms on pMOL30 resulted in a much greater sensitivity to the Cu stress. However, viable counts increased again after 144 h. This increase appeared at different levels and only in two out of the three replicates (Supplementary Figure 3). It is possible that mutations or genomic rearrangements occurred in these replicates that relieved the Cu stress, as this has previously been shown for zinc stress (Vandecraen et al., 2016), but this was not further examined by retesting or genome sequencing. In the presence of stainless steel, there is a small but non-significant decrease in viable counts. However, this decrease is far less drastic than that seen in the Cu condition. In the no-plate control condition, only a small decrease in viable counts was observed over the course of the experiment, which could again be ascribed to starvation and a slow conversion to the VBNC state. We did observe significant differences between the control conditions of the survival experiments with CH34 and AE104, starting after 96 h. Nevertheless, it is clear by the differences in viable counts of CH34 in the control of the Cu plate and ions experiments (see Effect of Cu Ions on Viable Counts, *p* < 0.05 after 168 h), which could be seen as biological replicates, that variation not related to the tested conditions is observed (Figures 1A, 5). Flow cytometry showed a significant decrease in the non-permeable cell fraction only in the presence of Cu from 24 h onward (Figure 1D). These results were comparable with those from strain CH34 (Figures 1C,D).

In a second experiment, the *C. metallidurans* CH34 cells were pre-induced with a non-lethal concentration of Cu^2+^ (300 μM). This concentration was previously shown to induce the cellular production of Cu resistance mechanisms (Monchy et al., 2006). Viable counts showed an initial small decrease after 5 h in the Cu condition (Figure 1E). Interestingly, they recovered completely after 48 h. It is clear that the initial stress imposed by the Cu plate on the pre-induced cell population elicits a smaller effect than that on the non-induced population. The decrease in viable counts for the pre-induced cells could putatively be ascribed to a difference in the Cu concentration experienced by the bacterial cells, combined with the difference in medium, from MM284 to mineral water, and the decrease in cell density, from 10^9^ CFU/ml to 10^8^ CFU/ml. While the Cu ion is predominantly present in its cupric form in both MM284 and mineral water (Cuppett et al., 2006), the concentrations of most anionic ligands are decreased in the latter, which could lead to a difference in the Cu-ligand species apparent to the bacterial cells. Though the initial viable counts are never reached again, the pre-induced *C. metallidurans* CH34 cells were better equipped to deal with the presence of the Cu plate. Interestingly, after 48 h the viable counts decreased again, reaching at the end of the experiment a number similar to the non-induced *C. metallidurans* CH34 cells. We hypothesize that the metabolic burden posed by the constant necessity for functional Cu resistance mechanisms cannot be sustained on longer terms by either stored energy reserves or the limited AOC present in the medium. In the stainless steel condition and control condition, no noticeable decrease was observed (Figure 1E). Flow cytometry data corresponded well with viable counts and the number of events in the non-permeable zone in the Cu condition is consistently and significantly higher than in either non-induced *C. metallidurans* CH34 or AE104 (Figures 1B,D,E; *p* < 0.05 for all samples between 48 h and 96 h).

### Biofilm Formation on Metallic Cu and Stainless Steel

**FIGURE 3:**
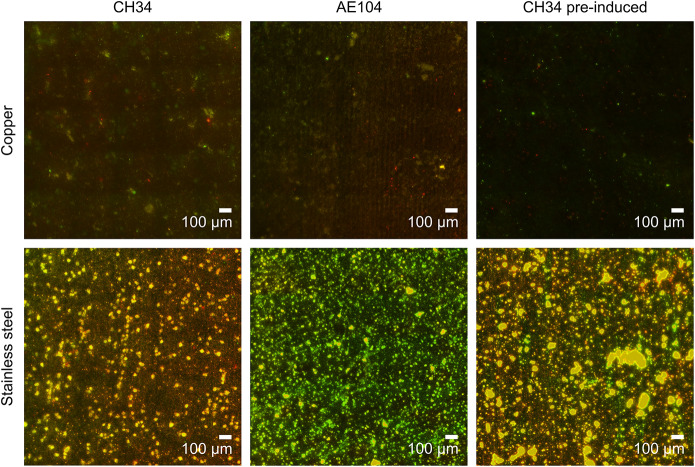
Biofilm formation of *C. metallidurans* CH34 (left column), *C. metallidurans* AE104, lacking metal resistance mechanisms (middle column), and *C. metallidurans* CH34 pre-induced with 300 μM CuSO_4_ (right column) on copper (top row) and stainless steel (bottom row). Cells were stained with SYBR Green and propidium iodide, and visualized by fluorescence microscopy.

At the end of the experiment (216 h after start), biofilms were visualized using fluorescence microscopy. Extensive biofilms were formed by all strains on stainless steel plates (Figure 3). In contrast, in none of the experiments with either non-induced *C. metallidurans* CH34, AE104, or pre-induced *C. metallidurans* CH34 cells, biofilms could be detected on the Cu plates. Some single cells were found on the surface of some plates, but these could be artifacts of an insufficient rinsing procedure. This result again showed the high toxicity of metallic Cu, even to the highly metal-resistant *C. metallidurans*. Since the toxicity of metallic copper also relies on the release of copper ions from the surface (Molteni et al., 2010), it is plausible that the copper resistance mechanisms of CH34 play a role in survival both in the bulk liquid phase and in close proximity to the metal surface. Our results do not refute the capacity of CH34 to form biofilms on the copper surface after longer incubation times and continued activation of its copper resistance mechanisms. Biofilm formation on copper pipes after long incubation times has already been observed, even for less metal-resistant bacterial species (Lehtola et al., 2004; Yu et al., 2010; Sarandan et al., 2011; Jungfer et al., 2013).

### Cu Concentrations in the Liquid Phase

**FIGURE 4:**
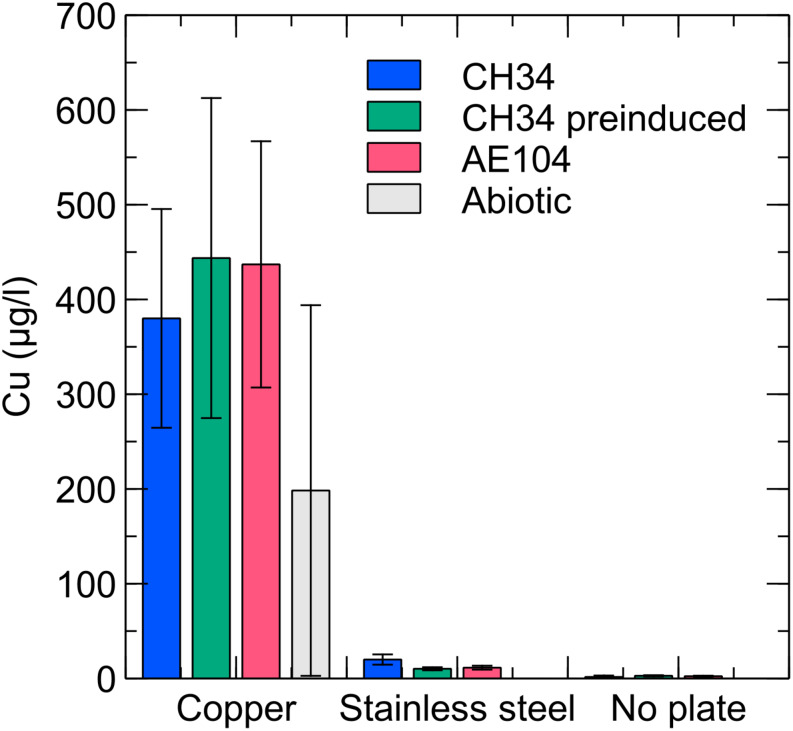
Liquid phase Cu concentrations for *C. metallidurans* CH34 (blue), *C. metallidurans* AE104, lacking metal resistance mechanisms (red), *C. metallidurans* CH34 pre-induced with 300 μM CuSO4 (green), and an abiotic control (gray) incubated for 9 days in the presence of Cu, stainless steel or no metal (control).

Copper concentrations were measured in the liquid phase by ICP–MS at several time points. In the copper condition with CH34, pre-induced CH34 and AE104 the copper concentration gradually increased, reaching ca. 400 μg/l (6.3 μM) at the end of the experiment (Figure 4). In addition, ICP–MS measurements confirmed the absence of residual Cu after washing the pre-induced CH34 cells (data not shown). The observed Cu concentrations were below the solubility of 1300 μg/l in mineral water at comparable pH (Cuppett et al., 2006) and far below the MIC of 3 mM in MM284 (Monsieurs et al., 2011). However, values were comparable to those applied in the previously mentioned studies, i.e., 1.6 μM, where a similar rapid decrease in viable counts was observed. Therefore, we hypothesize that in our experiments the toxic effects of Cu in the liquid phase play an important role, next to the possible contribution of contact killing, in the observed decrease in viable counts. We propose that there could be a net transport of Cu ions from the metal plate surface to the bacterial cells and that there is likely a high level of Cu sequestration by the cells, even considering the relatively low Cu concentrations in the liquid phase, because of the homogenization of concentration boundary layers by continuously shaking and a favorable association between Cu ions and bacterial cells (Santo et al., 2012). This cellular Cu sequestration may result in considerable stress for the bacteria. The Cu concentration in the abiotic control condition (i.e., without the addition of bacterial cells) is on average lower than in the three biotic conditions, although not statistically significant. Therefore, the perceptible enhanced release of Cu ions from the copper plate by bacterial interactions is not statistically justified. It has previously been shown that the presence of bacteria does not result in a higher rate of Cu ion dissolution from metal surfaces (Mathews et al., 2013). In the stainless steel and no plate conditions, very little Cu was present in the medium, approaching the detection limit of the measuring device (2 μg/l–0.03 μM). The presence of Cr and Ni, the main non-iron constituents in stainless steel, was not detected in any of the stainless steel samples.

### Effect of Cu Ions on Viable Counts

**FIGURE 5:**
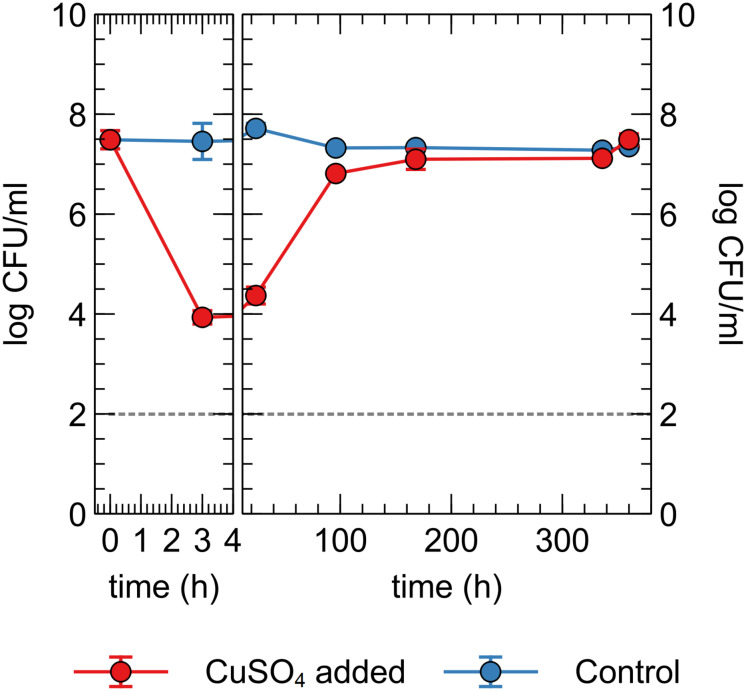
Viable counts of *C. metallidurans* CH34 in drinking water supplied with or without 10 μM CuSO_4_.

Next, we tested whether the observed decrease in viable counts in the Cu plate condition was due to a toxic effect of Cu ions in the liquid phase or close association with the Cu plate surface itself, via so-called “contact killing.” A CH34 suspension was prepared similarly to the first metal plate experiment, without pre-induction of Cu resistance mechanisms. Copper sulfate was added to the drinking water, to a final concentration of 10 μM (in range with the concentration observed in the liquid phase of the Cu plate experiments). Viable counts were measured at several time points for 15 days (Figure 5). Immediately apparent was the approximate 3-log decrease in viable counts after 3 h in the Cu condition, which could not be seen in the control condition. By 96 h, viable counts in the Cu condition were almost completely restored to control levels. While a similar rapid decrease in viable counts after the addition of Cu ions is seen in many bacterial species, the subsequent recovery or regrowth without addition of a chelating agent is not (Dwidjosiswojo et al., 2011; Jiang et al., 2016; Kan et al., 2019). When comparing this data to the Cu plate experiment data, it seems that the induction of Cu resistance mechanisms plays an important role in this setup as well, allowing for a full return to control condition levels. In contrast, the gradual decrease in viable counts observed in the Cu plate experiment after 144 h was not seen here, indicating a toxic effect either via contact killing or the gradual release of Cu ions from the metal plate as the closed system strives toward a chemical equilibrium.

### Influence of Medium Composition on the Induction of Cu Resistance Mechanisms

As demonstrated above, the presence of Cu resistance mechanisms plays a prominent role in the survival of bacterial cells when in wet contact with metallic Cu. However, despite the induction of these resistance mechanisms, cells are still being killed or inactivated as shown by the viable counts and flow cytometry. Therefore, we hypothesize that this induction is hampered by the low amount of nutrients and energy sources available.

To test this hypothesis the Cu biosensor strain *C. campinensis* AE1239, which is closely related to *C. metallidurans* CH34, was used. It emits a bioluminescent signal upon induction of Cu resistance genes. In a first experiment, we measured the bioluminescence of an AE1239 suspension on Cu and stainless steel plates (Supplementary Figure 4). Since Cu ions dissolved rapidly and massively when the Cu plates were placed in MM284 growth medium, this could not be used as a positive control. Therefore, the setup was first optimized on a stainless steel plate using a cell suspension in MM284 with CuSO_4_ added to a final concentration of 100 μM. This clearly yielded an induction with a maximal intensity reached around 220 min after the start of the induction. Without CuSO_4_, no induction was measured. When washed *C. campinensis* AE1239 cells were resuspended in sterile mineral water and pipetted onto a stainless steel plate, no induction was measured with CuSO_4_ concentrations of 0, 10, or 100 μM. When a cell suspension in sterile mineral water was pipetted onto a Cu plate surface, again no induction was measured within the 4 h of measurement time.

In a second setup we tested the effect of different CuSO_4_ concentrations on the induction of Cu resistance mechanisms in MM284 and sterile mineral water supplemented with varying levels of gluconate and Na_2_HPO_4_ (Supplementary Figure 5). A clear response to the Cu stress was visible in MM284 with 10 and 100 μM CuSO_4_, with a maximum induction at 150 min after Cu addition. Interestingly, irrespective of the addition of CuSO_4_, gluconate or Na_2_HPO_4_, no induction was observed in mineral water. It seems that additional nutrients are necessary to generate a measurable response to Cu stress, other than a source of cellular energy.

## Conclusion

We evaluated the inactivation kinetics of *C. metallidurans* CH34 in contact with metallic Cu in drinking water. While a rapid decrease in viable counts was observed within several hours, this is likely the result of a conversion to an injured or VBNC state. After several days, viable counts returned to the control level, an observation that has not been made in other bacterial species. This is likely due to a slow induction of Cu resistance mechanisms, as shown by the lack of recovery/regrowth of the AE104 strain (cured from most Cu resistance determinants) and the rapid recovery/regrowth of a CH34 culture with pre-induced resistance mechanisms. Based on similar inactivation kinetics in a setup supplemented with ionic Cu, we surmise that the toxic effect of Cu ions in the liquid phase plays a more important role than so-called “contact killing.” These results warrant caution in interpreting the growing body of evidence highlighting the antimicrobial properties of metallic Cu, especially in the context of drinking water production and storage (Vincent et al., 2016). Metal resistant bacterial strains, many among the genus *Cupriavidus*, have been isolated from water sources on many occasions (Manaia et al., 1990; Pindi et al., 2013; Berthiaume et al., 2014). During space missions, where the antimicrobial properties of metallic Cu are being evaluated to allow long-term inactivation of persistent microbial contamination, *C. metallidurans* strains have been isolated from drinking water in several sampling campaigns (Pierson, 2001; Baker and Leff, 2004; Ott et al., 2004). Since the induction of Cu resistance provides a clear advantage in surviving on or near metallic Cu, it raises the question to what extent resistance determinants can be transferred to or protect other bacterial species. In addition, co-selection between metal resistance and antibiotic resistance has often been reported (Baker-Austin et al., 2006; Stepanauskas et al., 2006; Dickinson et al., 2019; Nguyen et al., 2019). One way to amplify the killing efficiency of Cu metal could be to limit the extant AOC levels, that the bacteria need to synthesize and energize those resistance determinants. A final question that needs further research, here as well as in many similar publications (reviewed in Bogosian and Bourneuf, 2001), is in what ways the resuscitation of sublethally injured and VBNC cells is distinct from the regrowth of a small fraction of survivor cells.

## Data Availability Statement

All datasets presented in this study are included in the article/Supplementary Material.

## Author Contributions

LM, J-YM, and RV contributed conception and design of the study. LM and IC performed the experimental work. LM and JC performed the data analyses. LM wrote the first draft of the manuscript. All authors contributed to manuscript revision, read and approved the submitted version.

## Conflict of Interest

The authors declare that the research was conducted in the absence of any commercial or financial relationships that could be construed as a potential conflict of interest.
