# Supplementary material for: Copper Resistance Mediates Long-Term Survival of Cupriavidus metallidurans in Wet Contact With Metallic Copper
Source: Front Microbiol. 2020 Jun 3;11:1208. doi: 10.3389/fmicb.2020.01208 (PMC7284064; doi:10.3389/fmicb.2020.01208)
Supplement: Supplementary file 1 [file Data_Sheet_1.DOCX]

Supplementary Material


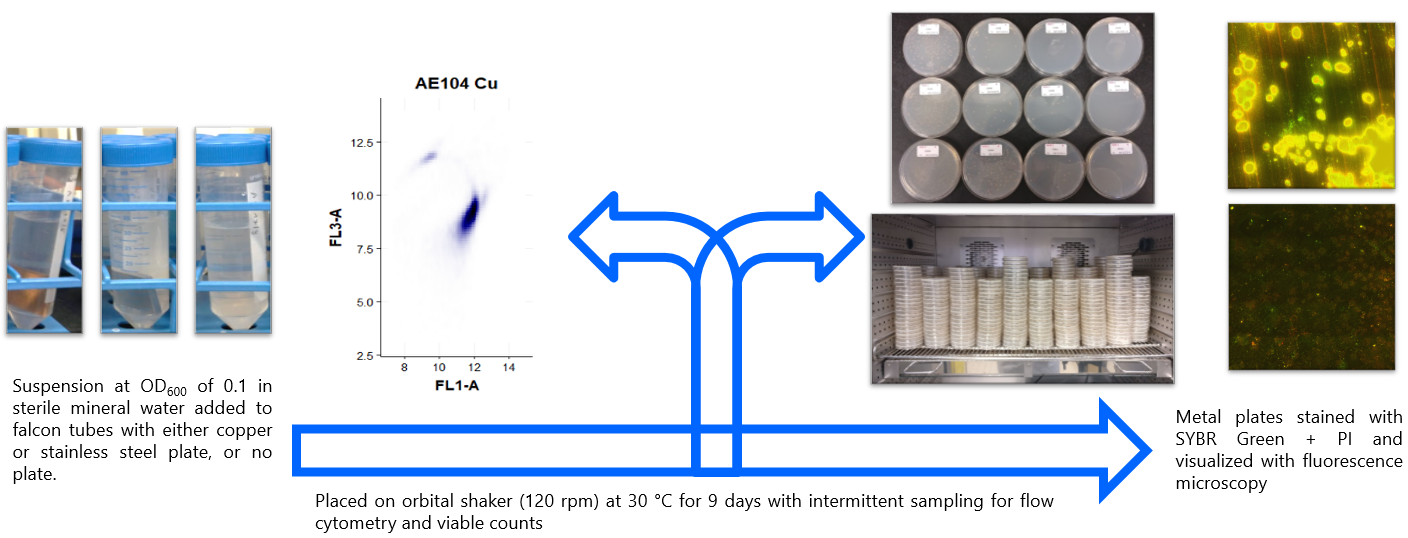


Supplementary Figure 1: Generalized overview of survival experiments with metal plates.


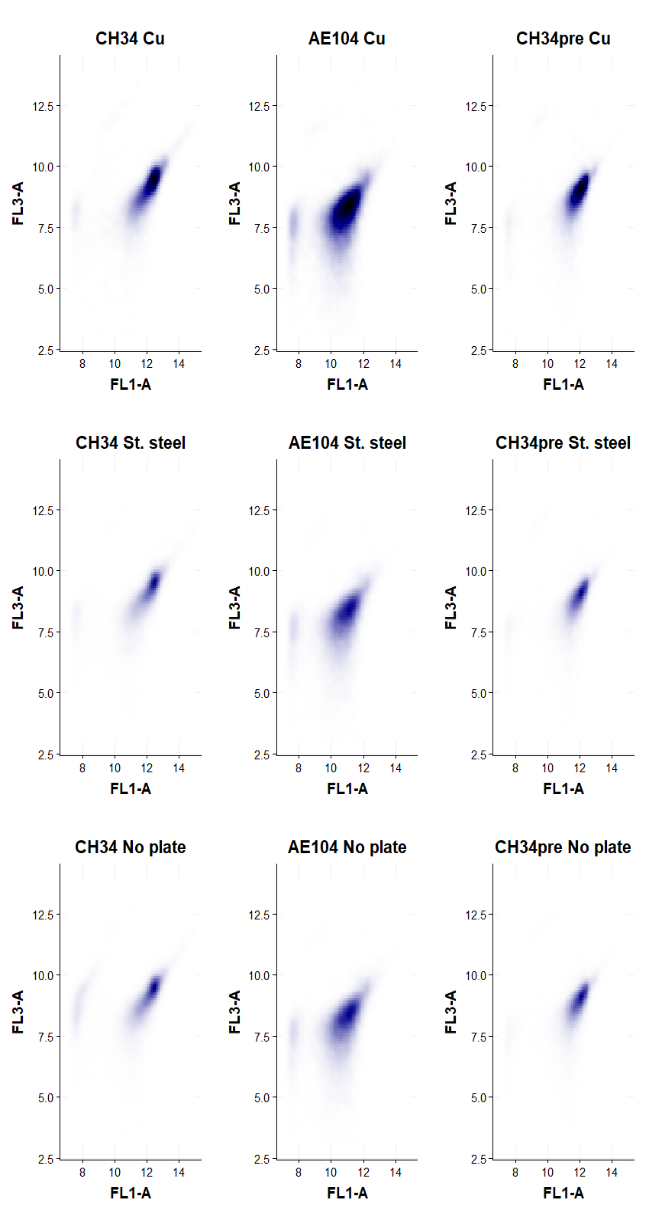

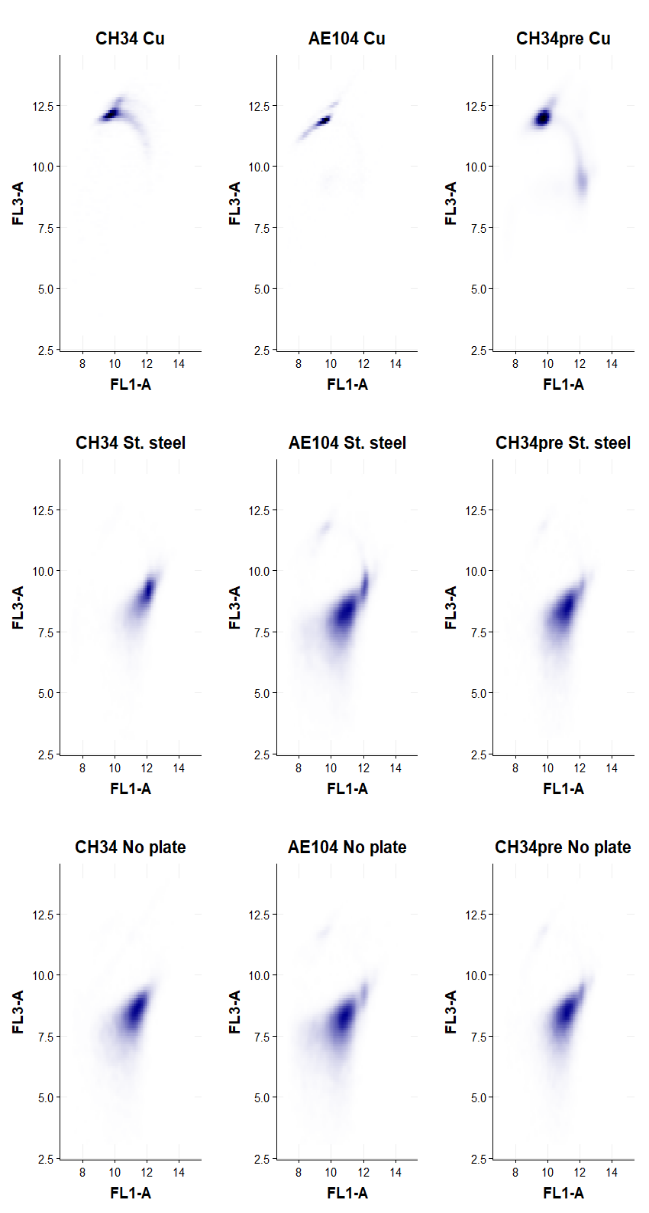

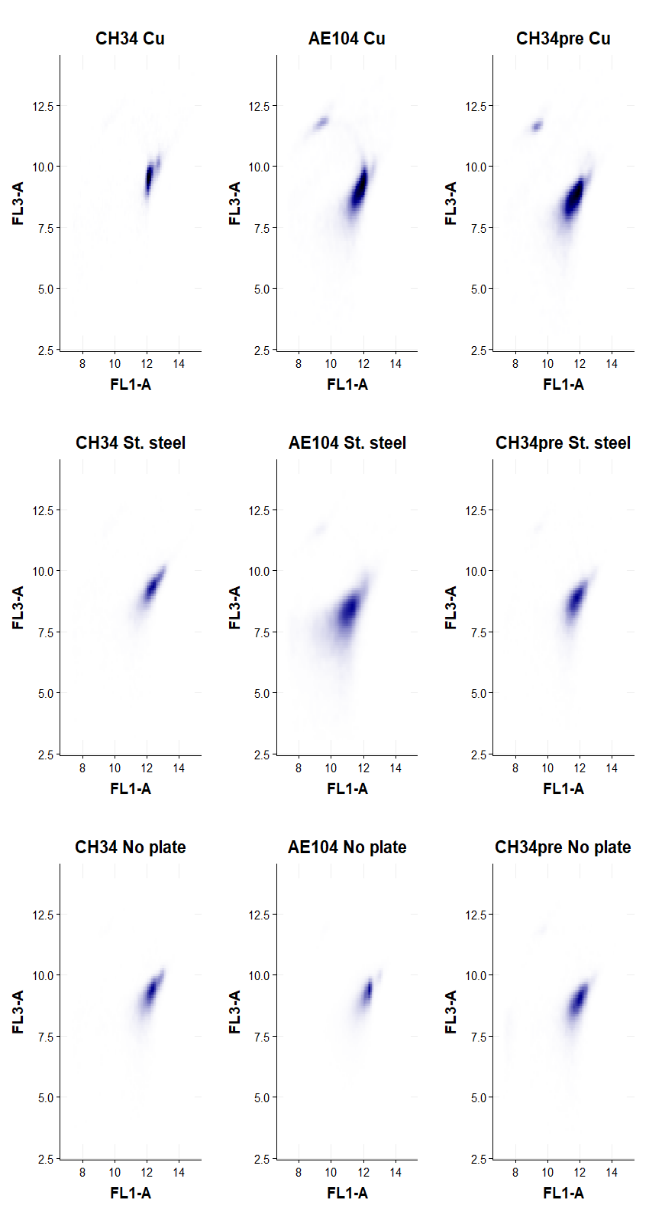
**0 h 5 h 48 h**

Supplementary Figure 2: Flow cytometric profiles (FL3-A ~ FL1-A, representing propidium iodide signal vs. SYBR Green signal) of cells in different conditions on three time points: 0 h (left), 5 h (middle), and 48 h (right). Within each time point: copper condition (top row), stainless steel condition (middle row), no plate control condition (bottom), *C. metallidurans* CH34 (left column), *C. metallidurans* AE104, lacking metal resistance mechanisms (middle column), *C. metallidurans* CH34 pre-induced with 300 µM of CuSO_4_ (right column). Green and red circles represent events corresponding to non-permeable and permeable cells, respectively.


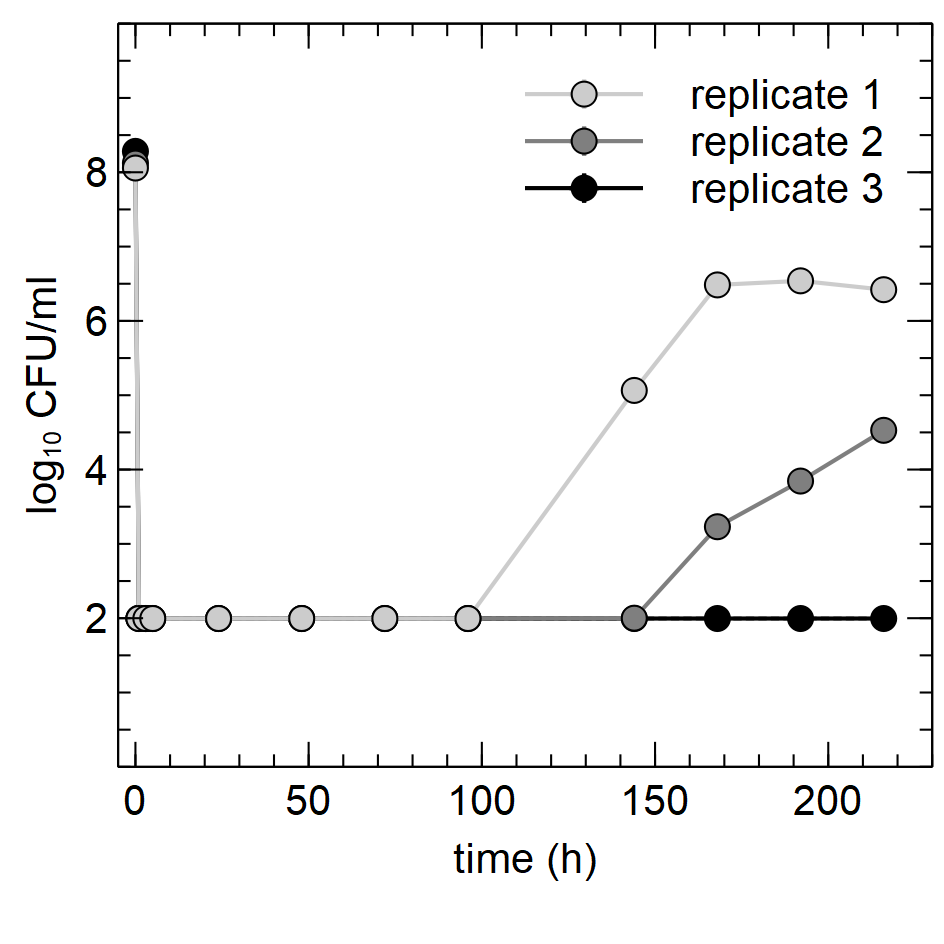


Supplementary Figure 3: Viable counts of *C. metallidurans* AE104, lacking metal resistance mechanisms, incubated in drinking water containing a copper metal plate.


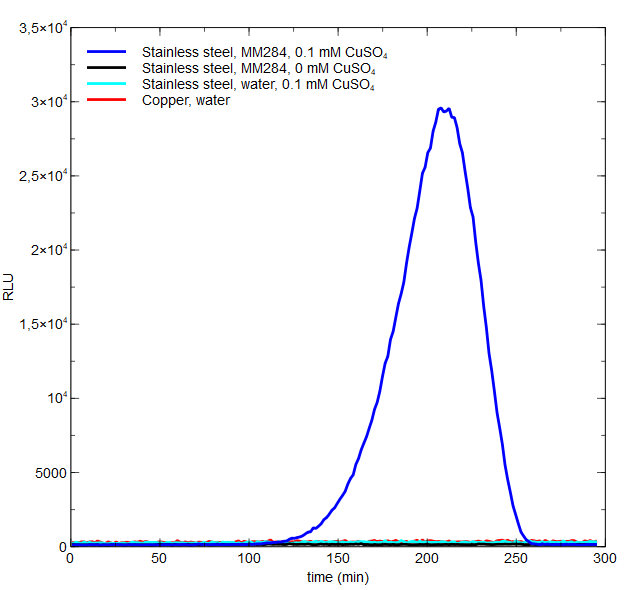


Supplementary Figure 4: Bioavailable Cu^2+^ concentration, measured by biosensor *C. campinensis* AE1239, in MM284 medium containing a stainless steel plate (black) supplemented with 0.1 mM CuSO_4_ (blue), in water containing a stainless steel plate and 0.1 mM CuSO4, and in water containing a copper plate. The bioluminence output of the reporter was recorded for 300 min and shown as relative light units (RLU).


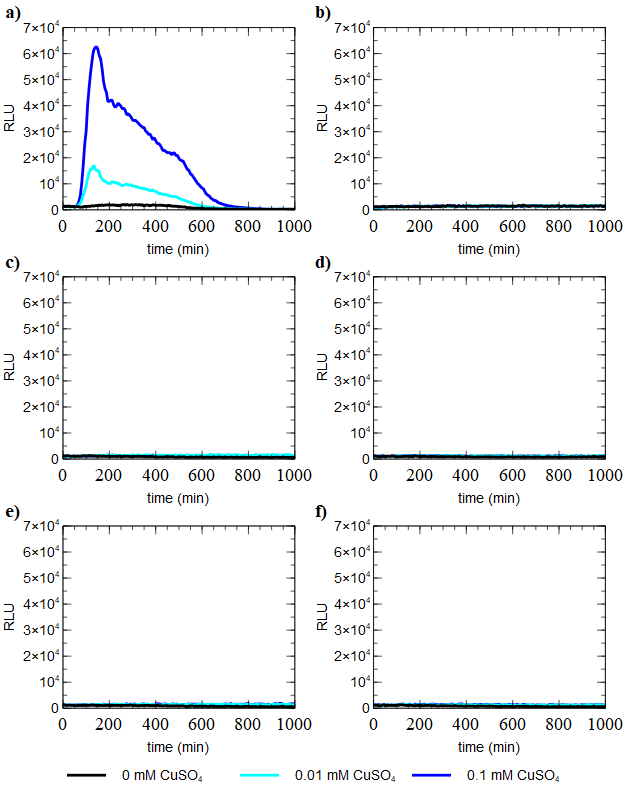


Supplementary Figure 5: Bioavailable Cu^2+^ concentration, measured by biosensor *C. campinensis* AE1239 in liquid media in 6 conditions: MM284 (a) filter sterilized drinking water (b) drinking water supplemented with 0.2 g/l Na-gluconate added (c) drinking water supplemented with 0.2 g/l Na_gluconate and 40 mg/l Na_2_HPO_4_ added (d) drinking water supplemented with 2 g/l Na-gluconate (e) drinking water supplemented with 2 g/l Na-gluconate and 40 mg/l Na_2_HPO_4_ (f). The bioluminence output of the reporter was recorded for 1000 min and shown as relative light units (RLU) normalized by cell density (OD_600_).aa a
